# Focal disruption of DNA methylation dynamics at enhancers in IDH-mutant AML cells

**DOI:** 10.1038/s41375-021-01476-y

**Published:** 2021-12-06

**Authors:** Elisabeth R. Wilson, Nichole M. Helton, Sharon E. Heath, Robert S. Fulton, Jacqueline E. Payton, John S. Welch, Matthew J. Walter, Peter Westervelt, John F. DiPersio, Daniel C. Link, Christopher A. Miller, Timothy J. Ley, David H. Spencer

**Affiliations:** 1 Department of Medicine, Division of Oncology, Section of Stem Cell Biology, Washington University, St. Louis, MO USA; 2 Department of Pathology and Immunology, Washington University, St. Louis, MO USA; 3 McDonnell Genome Institute, Washington University, St. Louis, MO USA

**Keywords:** Acute myeloid leukaemia, Cancer genomics

## Abstract

Recurrent mutations in *IDH1* or *IDH2* in acute myeloid leukemia (AML) are associated with increased DNA methylation, but the genome-wide patterns of this hypermethylation phenotype have not been comprehensively studied in AML samples. We analyzed whole-genome bisulfite sequencing data from 15 primary AML samples with *IDH1* or *IDH2* mutations, which identified ~4000 focal regions that were uniquely hypermethylated in *IDH*^*mut*^ samples vs. normal CD34+ cells and other AMLs. These regions had modest hypermethylation in AMLs with biallelic *TET2* mutations, and levels of 5-hydroxymethylation that were diminished in *IDH* and *TET*-mutant samples, indicating that this hypermethylation results from inhibition of TET-mediated demethylation. Focal hypermethylation in *IDH*^mut^ AMLs occurred at regions with low methylation in CD34+ cells, implying that DNA methylation and demethylation are active at these loci. AML samples containing *IDH* and *DNMT3A*^R882^ mutations were significantly less hypermethylated, suggesting that *IDH*^mut^-associated hypermethylation is mediated by DNMT3A. *IDH*^mut^-specific hypermethylation was highly enriched for enhancers that form direct interactions with genes involved in normal hematopoiesis and AML, including *MYC* and *ETV6*. These results suggest that focal hypermethylation in *IDH*-mutant AML occurs by altering the balance between DNA methylation and demethylation, and that disruption of these pathways at enhancers may contribute to AML pathogenesis.

## Introduction

DNA methylation changes in acute myeloid leukemia (AML) are caused by disruptions in the processes that add or remove 5-methyl groups to cytosines (5mC) [1, 2]. In normal and malignant hematopoietic cells, de novo DNA methylation is catalyzed primarily by the DNA methyltransferase DNMT3A [3, 4], which methylates unmethylated DNA substrates. Demethylation occurs passively after DNA synthesis in the absence of DNMT1-mediated propagation of hemi-methylated DNA, and actively via hydroxylation of 5mC by the TET family of hydroxylases. Alterations in these opposing forces result in either increased or decreased DNA methylation in AML cells. These changes include diffuse hypomethylation across large genomic regions and focal hypermethylation in CpG islands (CGIs). We recently showed that CGI hypermethylation in AML is mediated by DNMT3A and is present in nearly all AML subtypes [5]. In addition to these changes, specific DNA methylation patterns correlate with AML mutations that influence DNA methylation. This includes the *DNMT3A*^R882^ mutation, which impairs DNA methylation activity and results in a focal, canonical hypomethylation phenotype [5].

Mutations in *IDH1* and *IDH2* are also associated with altered DNA methylation patterns [6, 7] that are thought to occur by disrupting active DNA demethylation. *IDH1* and *IDH2* encode metabolic enzymes not normally involved in DNA methylation, but when mutated produce 2-hydroxyglutarate (2HG) [8] that inhibits the TET family of enzymes [9], thereby reducing active demethylation. Analysis of DNA methylation in primary AML samples using array-based technologies and enhanced reduced-representation bisulfite sequencing has demonstrated that DNA methylation is increased in samples with *IDH* mutations [6, 10]. While the direct effects of these changes on gene regulation have been challenging to identify, the contribution of *IDH* mutations to leukemogenesis has been established in mouse models. Expression of either *IDH1*^R132H^ or *IDH2*^R140Q^ blocks normal hematopoietic differentiation, promotes myeloproliferation [11–13], and can result in AML transformation in the presence of cooperating mutations [13, 14]. These studies establish the contribution of *IDH* mutations to AML development and suggest this may occur by disrupting the balance between DNA methylation and demethylation.

Although previous studies using targeted DNA methylation approaches have reported the general effects of *IDH1* and *IDH2* mutations on DNA methylation [6, 7, 10, 15], genome-wide methylation analysis in primary AML samples has not yet been described. It is therefore unclear whether *IDH1* vs. *IDH2* mutations cause hypermethylation at the same or different genomic loci, and whether these methylation changes are distinct from DNMT3A-mediated CGI hypermethylation. In addition, although *IDH* mutations are thought to cause hypermethylation via inhibition of TET enzymes, the overlap in methylation phenotypes between AML samples with these mutations is unclear. Here, we performed a genome-wide analysis of DNA methylation in primary AML samples with recurrent mutations in *IDH1*, *IDH2*, or *TET2* using whole-genome bisulfite sequencing (WGBS). WGBS data from normal hematopoietic cells and AML samples with other mutational profiles were included to define the methylation phenotypes specific to *IDH* mutations and to determine their relationship to “generic” AML-associated methylation changes. We integrated these data with epigenetic modifications and three-dimensional (3D) genome architecture from primary AML samples to characterize the functional genomic elements that may be affected by disruption of the balance between DNA methylation and demethylation in AML.

## Materials and methods

### Patient samples

Primary AML samples and normal hematopoietic cells for epigenetic studies were obtained from presentation AML and normal bone marrow aspirates, following informed consent using the protocol (201011766) approved by the Human Research Protection Office at Washington University as described previously [5] (Table S1). All experiments with AML samples used total bone marrow cells for DNA preparation.

### Whole-genome bisulfite and oxidative bisulfite sequencing and data analysis

WGBS data for 38 samples were described previously [5]. Data for 13 additional samples were generated using 50 ng of DNA with the Swift Accel-NGS Methyl-Seq library preparation kit. Oxidative bisulfite sequencing libraries were prepared following treatment of 200 ng of DNA with the TrueMethyl oxBS module (Cambridge Epigenetix) prior to bisulfite conversion and Swift library construction and sequencing on NovaSeq 6000 instruments (Table S1). Data were aligned to the GRCh38 reference and processed into methylated read counts using biscuit [16] with default parameters. Differentially methylated CpGs (DMCs) were identified between AML groups and CD34+ cells using read count data via DSS [17] and required a minimum methylation difference of 0.2. DMCs were then used to identify differentially methylated regions (DMRs) with >10 CpGs and a difference in mean methylation of 0.2. *IDH*^mut^- and *TET2*^mut^-specific DMCs and DMRs were subsequently identified by comparing these samples to all other AML samples via the DSS beta-binomial test in the methylkit Bioconductor package [18]. 5hmC values were obtained by subtracting the methylation ratios from OxWGBS data from WGBS data at all CpGs with coverage >10×.

### ChIP-seq for histone modifications

ChIP-seq was performed using ChIPmentation [19] with the following antibodies: H3K27me3 (9733 S), and H3K27ac (8173 S) from Cell Signaling Technology, and H3K4me1 (ab1012) from Abcam. Sequencing was performed on a NovaSeq 6000 (Illumina, San Diego, CA) to obtain ~50 million 2 × 150 bp reads. Data were analyzed via adapter trimming with trimgalore and alignment to GRCh38 using bwamem [20]. Normalized coverage for visualization and analysis used the deeptools “bamCoverage” tool [21], and peaks were called with MACS2 [22]. Statistical comparisons with DESeq2 [23] used raw fragment counts at peak summits, and visualizations were prepared with Gviz [24]. Superenhancer analysis was conducted using ROSE software [25, 26] with default parameters.

### RNA-seq analysis

RNA-seq data from AML samples were obtained from the AML TCGA study [15]. TPM values were obtained using kallisto [27] and gene counts were generated using the tximport Bioconductor package [28] in R with the tx2gene option set to accomplish gene-level summarization. Previously published RNA-seq data for normal CD34+ cells generated using the same procedures that were used for the AML samples [29, 30] were obtained as raw sequencing reads from the short-read archive (GSE48846) and processed as described above.

### HiC data analysis

HiC data were obtained from previous studies of 3D genome interactions in primary AML samples [31] and normal hematopoietic stem/progenitors [32]. All libraries were generated using MboI digestion prior to proximity ligation and data were analyzed using the juicer pipeline [33]. Loops were identified with HICCUPs and were analyzed using bedtools [34] to identify overlap with genes and putative enhancers. Visualizations used the GenomicInteractions and Gviz R packages [24].

## Results

### Primary AML samples with *IDH1* or *IDH2* mutations are focally hypermethylated at regions with low methylation in normal hematopoietic cells

**Fig. 1:**
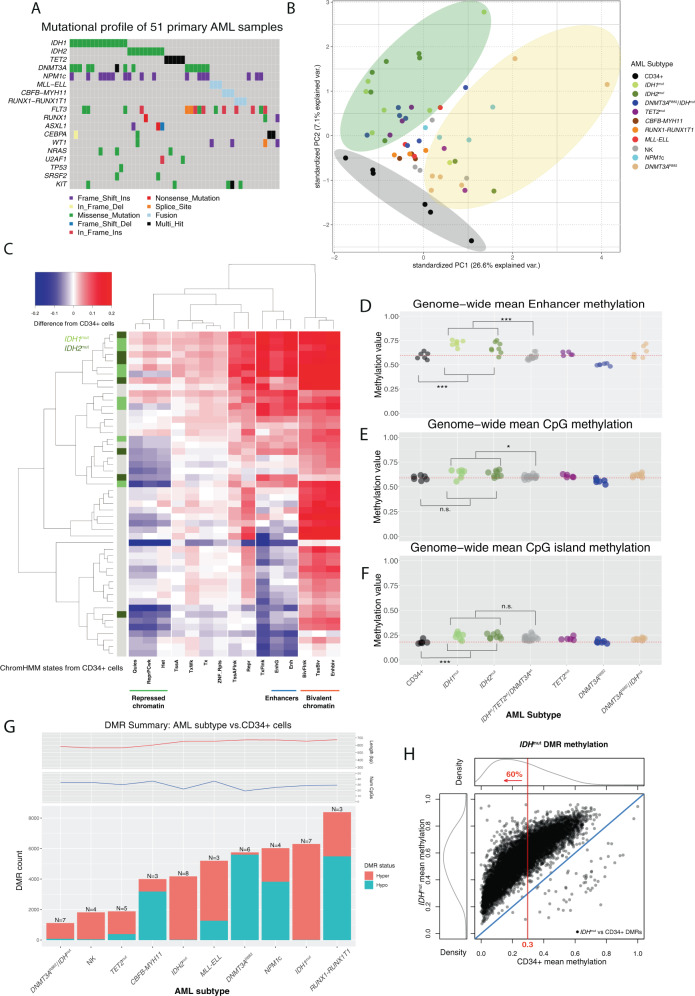
Genome-wide DNA methylation patterns in 51 primary AML samples and normal CD34+ cells. **A** Summary of the mutations in 51 primary AML patients analyzed using whole-genome bisulfite sequencing. **B** Principal component analysis of genome-wide methylation in AML samples and CD34+ cells. Points show the values of the first and second principal components by variance explained from an analysis of genome-wide methylation summarized in mean methylation in 1 kb bins, with colors representing the defining mutation for each sample. Stratification of *IDH*^mut^ samples from CD34+ cells and AML samples with *DNMT3A*^R882^ mutations are highlighted by colored ellipses. **C** Two-way hierarchical clustering of relative (difference from CD34+ cells) mean methylation levels in genomic regions defined by 15 chromatin states [36] in CD34+ cells, where rows are AML samples and columns are chromatin states. Blue is less methylated than CD34+ cells and red is more methylated. *IDH1* and *IDH2* mutation status are indicated in the colored bar on the left, and selected chromatin states are shown underneath the panel. **D** Mean methylation levels in the enhancer chromHMM state (derived from publicly available CD34+ epigenetic data) from WGBS for CD34+ cells (*N* = 6) and AML subtypes (*IDH1*^mut^ or *IDH2*^mut^, *n* = 15; *TET2*^mut^, *n* = 5; *DNMT3A*^R882^, *n* = 6; *DNMT3A*^R882^/*IDH*^mut^, *n* = 7; normal karyotype with *NPM1c* and wild-type *IDH1*, *IDH2*, *TET2*, and *DNMT3A*, *n* = 4; Normal karyotype with wild-type *NPM1*, *IDH1*, *IDH2*, *TET2*, and *DNMT3A*, *n* = 4; *CBFB-MYH11*, *n* = 3; *MLL-ELL*, *n* = 3; *RUNX1-RUNX1T1*, *n* = 3). **E** Mean methylation levels for ~28 million genome-wide CpGs in CD34+ cells and AML subtypes. **F** Mean methylation at CpG islands in CD34+ cells and AML subtypes. **G** Number of differentially methylated regions (DMRs) identified for each AML subtype compared with normal CD34+ cells. Teal and orange bars represent hypomethylated and hypermethylated DMRs with respect to normal CD34+ cells, respectively. Mean number of CpGs per DMR (top panel) and DMR length (bottom panel) are shown for each AML subtype. **H** Mean methylation in *IDH*^mut^ DMRs in *IDH*^mut^ samples versus CD34+ cells. The red line indicates the percent of all DMRs with mean methylation in CD34+ cells <0.3.

We performed WBGS using 15 primary bone marrow aspirate samples from AML patients with canonical *IDH* mutations, including seven with *IDH1*^R132C/G^, seven with *IDH2*^R140Q^, and one with an *IDH2*^R172K^ allele (referred to hereafter as *IDH*^mut^). These data were analyzed with WGBS data from 36 other primary AML samples representing nine mutational categories, including five with biallelic loss-of-function mutations in *TET2*, and primary CD34+ cells from six healthy adults bone marrow donors [5]. All AML samples were previously sequenced using whole-genome and/or whole-exome sequencing [15, 35] that confirmed the mutations affecting DNA methylation were present in the dominant leukemic clone (Fig. 1A). Importantly, the 15 AML samples with *IDH* mutations were wild type for *DNMT3A* and *TET2* to minimize the effects of other mutations on DNA methylation patterns. We first performed an unsupervised analysis of genome-wide methylation in 1 kb bins using principal component analysis. This demonstrated that most AML samples formed a diffuse cluster separate from CD34+ cells (Fig. 1B). AML samples with either *DNMT3A*^R882^ or *IDH* mutations (and some with *TET* mutations) formed sub-clusters on opposite sides of the main AML group, which is consistent with the hypomethylation phenotype of AML cells with the *DNMT3A*^R882^ mutation [5] and suggests *IDH*^mut^ samples may also have unique methylation features compared to other AMLs.

We next determined whether *IDH* mutations have global or context-dependent effects on DNA methylation by analyzing methylation levels in regions defined by chromatin states in hematopoietic stem/progenitors [36]. This demonstrated that quiescent and repressed chromatin states had lower methylation in most AMLs compared to CD34 cells, whereas bivalent regions (which are enriched for CGIs) were hypermethylated in nearly all samples (Fig. 1C). Enhancers and regions flanking transcriptional start sites (TSS) supervised a cluster of hypermethylated AMLs containing 14 of the 15 *IDH*^mut^ samples. Mean methylation in *IDH*^mut^ AMLs at enhancer regions was significantly higher vs. both CD34+ cells and AMLs without *IDH* mutations (Fig. 1D, *P* = 0.009 and *P* = 0.0002, respectively). *IDH*^mut^ AMLs also tended to have higher mean methylation vs. other AML groups both genome-wide (Fig. 1E, adjusted *P* = 0.02) and in regions with other chromatin states (Figs. S1A, B), but not in CGIs (Fig. 1F; *P* = 0.14), indicating that *IDH* mutations do not result in an exaggerated CGI hypermethylation phenotype.

We next determined the extent to which *IDH* mutations result in focal methylation changes by performing differential methylation analysis [37] between AMLs with *IDH1* or *IDH2* mutations and CD34 cells. There were 6309 differentially methylated regions (DMRs) in *IDH1*^mut^ AMLs, of which 99% were hypermethylated relative to CD34+ cells (methylation difference >0.2, FDR < 0.05 with >10 CpGs; Fig. 1G); this was more than any mutation-defined AML group. *IDH2*^mut^ AMLs had fewer DMRs (*N* = 4195), although most were also hypermethylated (85%). AMLs with *IDH1* or *IDH2* mutations also had the highest fraction of hypermethylated CpGs (DMCs) (85% and 87%, respectively; see Fig. S1C), most of which were contained in DMRs (Fig. S1D, E). Interestingly, although *IDH* mutations are thought to inhibit active demethylation, most *IDH*^mut^ DMRs had low methylation in normal hematopoietic cells. For example, 60% of the *IDH*^mut^ DMRs had mean methylation <0.3 in both CD34+ cells (Fig. 1H) and more mature myeloid cell populations (Fig. S1F), suggesting that DNA methylation pathways must be active in these regions despite the low methylation levels at these loci in normal cells.

### *IDH*^mut^-specific methylation changes are distinct from AML-associated CGI hypermethylation and are influenced by *IDH* mutation type

**Fig. 2:**
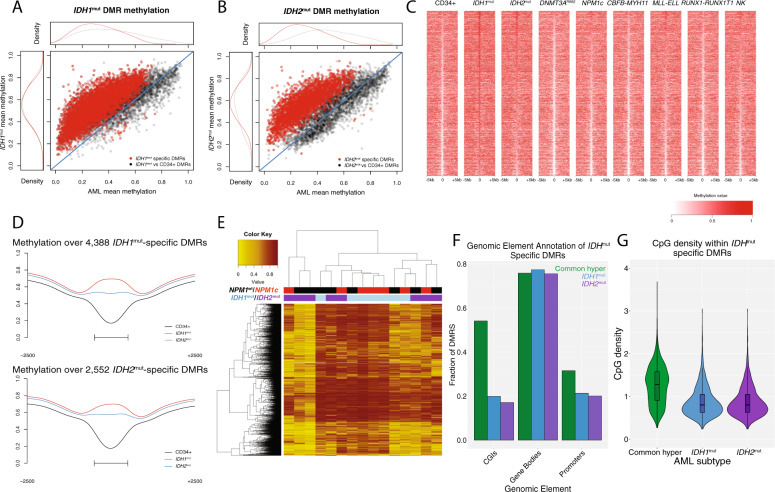
Characterization of *IDH*^mut^-specific DMRs. **A** Mean methylation in all *IDH1*^mut^ associated DMRs vs. CD34+ cells (*n* = 6309; black points) and in *IDH1*^mut^-specific DMRs that are unique compared with all other AMLs (*n* = 4388; red points). **B** Mean methylation in all *IDH2*^mut^ associated DMRs vs. CD34+ cells (*n* = 4195; black points) and in *IDH2*^mut^-specific DMRs that are unique compared to all other AMLs (*n* = 2552; red points). **C** Locus heatmap showing mean methylation values by mutation group for the union of *IDH1*^mut^ and *IDH2*^mut^-specific DMRs, where each column is centered over the DMR with the window extending 5 kb upstream and downstream of the DMR center. **D** Mean methylation across all *IDH1/2*^mut^-specific DMRs (rows) in 15 individual *IDH*^mut^ cases (columns). The mutation status of *IDH1*, *IDH2*, and *NPM1* are indicated by the colored bars above the heatmap. Note that all AML samples in this panel are wild type for *DNMT3A* and *TET2*. **E** Aggregate DMR methylation across 4388 *IDH1*^mut^ specific DMRs and 2552 *IDH2*^mut^ specific DMRs respectively. **F** Fraction of generically hypermethylated DMRs, *IDH1*^mut^-specific DMRs, and *IDH2*^mut^-specific DMRs overlapping functional genomic elements. **G** Distribution of CpG densities across generically hypermethylated regions in primary AML and *IDH1*^mut^-and *IDH2*^mut^-specific DMRs.

We next performed a second statistical comparison of the DMRs (and DMCs) identified in AMLs with *IDH* mutations vs. CD34+ cells to identify loci with methylation levels in the *IDH*^mut^ samples that were significantly different from all other AML samples. AMLs with mutations in *DNMT3A* or *TET2* were excluded from this analysis given their established hypomethylation phenotype (*DNMT3A*) and potential to phenocopy *IDH* mutations (*TET2*). This resulted in 4388 and 2552 *IDH1*^mut^ and *IDH2*^mut^-specific DMRs, respectively, nearly all of which were hypermethylated relative to the other AML samples (Fig. 2A–C, Tables S2, 3). Similar results were observed at the DMC level (Fig. S2A, B). Most of these DMRs displayed low methylation in normal cells, with 60% of *IDH1*^mut^-specific and 58% of *IDH2*^mut^-specific loci having a methylation level <0.3 in CD34+ and mature myeloid cells (Fig. S2C, D). There was extensive overlap between the *IDH* mutation-specific DMRs (94% [2399/2552] of *IDH2*^mut^-specific DMRs overlapped an *IDH1*^mut^-specific DMR), and AML samples with either mutation were hypermethylated at both DMR sets (Fig. 2D). However, hierarchical clustering demonstrated considerable variability in methylation between the *IDH1*^mut^ and *IDH2*^mut^ samples (Fig. 2E). Notably, three *IDH2*^mut^ AMLs had lower methylation across the union of *IDH*^mut^-specific DMRs (*N* = 4541). *IDH2*^mut^ AML samples were also less methylated than *IDH1*^mut^ samples at the combined set of *IDH*^mut^ DMRs (0.54 vs. 0.70, respectively; *P* = 0.04), but were hypermethylated relative to CD34+ cells (Fig. 2E). This was not related to mutant *IDH* allele abundance (all samples had VAFs >30%, Table S1), and did not correlate with other recurrent mutations, including *NPM1*c (four in *IDH1*^mut^ and 3 in *IDH2*^mut^ samples, Fig. 2D; all samples were wild type for *DNMT3A* and *TET2*). Comparable differences in methylation were observed at the DMC level (Fig. S2E, F), suggesting this phenomenon was not an artifact of DMR identification.

Interestingly, the *IDH*^mut^-specific DMRs demonstrated markedly different CpG density and overlap with genomic annotations compared to hypermethylated regions in other AML samples. For example, both *IDH1*^mut^-specific and *IDH2*^mut^-specific DMRs displayed significantly less overlap with annotated CGIs compared to 4573 hypermethylated regions identified in at least two other AML mutation categories (20% and 18% of *IDH1*^mut^ and *IDH2*^mut^ DMRs overlapped a CGI, respectively, compared to 54% of commonly hypermethylated regions; see Fig. 2F), and had lower CpG density (mean CpG density of 0.81 and 0.79 vs. 1.26, respectively; *P* values < 0.0001; Fig. 2G). Promoters were also underrepresented in *IDH1*^mut^-specific and *IDH2*^mut^-specific DMRs (21% and 20% of *IDH1*^mut^ and *IDH2*^mut^ DMRs overlapped a promoter, vs 31% of commonly hypermethylated regions; Fig. 2G). *IDH*^mut^-specific DMCs showed similar levels of overlap with annotated regions as DMRs (Fig. S2G), further suggesting that *IDH*-associated hypermethylation is distinct from AML-associated CGI hypermethylation.

### Hypermethylation in *TET2*^mut^ AMLs overlaps with *IDH*^mut^-specific hypermethylation, but does not phenocopy the extent of methylation changes

**Fig. 3:**
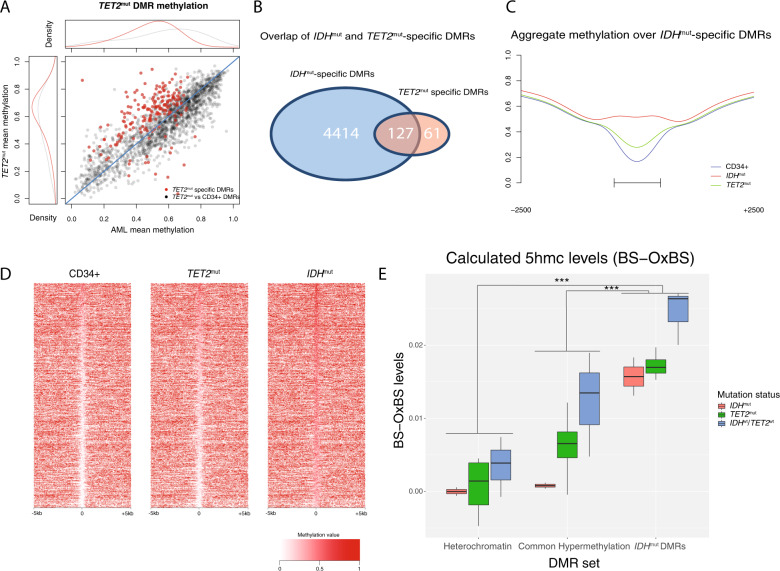
*TET2*^mut^ AMLs have modest hypermethylation that overlaps *IDH*^mut^-specific DMRs. **A** Mean DMR methylation across 1879 *TET2*^mut^ DMRs called vs. CD34+ cells (black points) and 188 in *TET2*^mut^-specific DMRs (red points) in *TET2* mutant samples versus all other AMLs that are wild type for *IDH, TET2, and DNMT3A*. **B** Intersection of *TET2*^mut^-specific and *IDH*^mut^-specific DMRs. **C** Aggregate methylation over *IDH*^mut^-specific DMRs in *IDH*^mut^ and in *TET2*^mut^ AML and CD34+ cells. **D** Locus heatmap of mean methylation values for all *IDH*^mut^-specific DMRs (rows), where each column is centered over the DMR with the window extending 5 kb up- and downstream the DMR center point. **E** Mean 5hmC (WGBS minus oxWGBS) levels in *IDH*^mut^, *TET2*^mut^, and *IDH*^wt^/*TET2*^wt^ AML samples at 105,519 ChromHMM heterochromatic regions, 4586 generically hypermethylated regions (i.e., regions that were hypermethylated vs. CD34+ cells in at least two AMLs without *IDH1*, *IDH2*, or *TET2* mutations), and 4541 *IDH*^mut^-specific hypermethylated DMRs.

We next determined whether AML samples with biallelic loss-of-function mutations in *TET2* shared similar genome-wide patterns of hypermethylation with *IDH*^mut^ AMLs. Initial comparison of the *TET2*^mut^ AMLs vs. normal CD34+ cells yielded fewer DMRs (and DMCs) and a lower proportion of hypermethylated regions compared to the combined set of DMRs in *IDH*^mut^ samples (1879 vs. 7569 DMRs, and 75% vs 99% hypermethylated regions, respectively; see Fig. 1G and S1A), consistent with previous reports [6, 10]. Hierarchical clustering of *TET2*^mut^ samples with the set of *IDH*^wt^/*TET2*^wt^/*DNMT3A*^wt^ AMLs at these regions did not reveal striking methylation differences between the two groups (Fig. S3A). Consistent with this result, only 188 *TET2*^mut^*-*specific DMRs were identified using the approach described above (with *IDH*^mut^ and *DNMT3A*^R882^ AMLs excluded from the analysis) (Fig. 3A). Although most *TET2*^mut^-specific DMRs were hypermethylated relative to CD34+ cells and other AMLs (171 of 188), the fraction was less than in either *IDH1*^mut^ or *IDH2*^mut^ AMLs (89% vs 99% and 99%, respectively). Similarly, *TET2*^mut^-specific DMCs showed subtle hypermethylation (Fig. S3B). *TET2*^mut^-specific DMRs were also not enriched for CGIs and promoters compared to a set of regions commonly hypermethylated in AML (12% of *TET2* DMRs overlapped a CGI vs. 54% of common hypermethylated DMRs; 17% of *TET2* DMRs overlapped a promoter vs. 31% of hypermethylated DMRs; see Fig. S3C, D), suggesting these regions do not reflect CGI hypermethylation.

To investigate the interaction between *IDH*^mut^ and TET2-mediated demethylation, we compared *TET2*^mut^-specific and *IDH*^mut^-specific DMRs and performed oxidative bisulfite sequencing [38] to measure 5-hydroxymethylation (5hmC) in *TET2*^mut^, *IDH*^mut^, and *TET2*^wt^/*IDH*^wt^ samples. This analysis showed that 68% (127 of 188) of the *TET2*^mut^-specific DMRs overlapped an *IDH*^mut^-specific hypermethylated region (Fig. 3B; *P* < 0.0001 using a permutation test for overlaps using all DMRs identified in any AML group). *TET2*^mut^ AMLs also displayed higher methylation levels at the combined set of 4541 *IDH*^mut^-specific DMRs compared to CD34+ cells (mean methylation of 0.35 vs. 0.26; 40% of DMRs with increased methylation via beta-binomial hypothesis testing with adjusted *P* < 0.05; Fig. 3C, D). Analysis of 5hmC using paired oxidative and standard whole-genome bisulfite sequencing (oxWGBS and WGBS with conversion rates ranging from 73–83%; see Fig. S3E) demonstrated low calculated levels of 5hmC across the genomes of all samples (0.44–0.66% in *TET2*^mut^, 0.52–1.22% in *TET2*^wt^, 0.17–0.25% in *IDH*^mut^; Fig. S3F), with higher levels in enhancer regions (Fig. S3G) and identifiable peaks at selected loci (Fig. S3H). Calculated 5hmC was statistically higher in *IDH*^mut^ DMRs compared to regions that were hypermethylated in other AML samples or in constitutively methylated heterochromatic regions (adjusted *P* = 0.0009 and *P* = 4 × 10^−7^ for a difference in mean 5hmC in all samples at *IDH*^mut^ DMRs vs. 4586 commonly hypermethylated DMRs and 105,519 heterochromatin regions, respectively; see Fig. 3E, S2I, J). AML samples with *TET2*, *IDH1*, or *IDH2* mutations had lower calculated 5hmC levels at *IDH*^mut^ DMRs compared to AMLs that were wild type for these genes (Fig. 3E, S2I, J), providing evidence that these mutations influence methylation turnover at these loci.

### DNA hypermethylation in *IDH*^mut^ AML cells requires DNMT3A

**Fig. 4:**
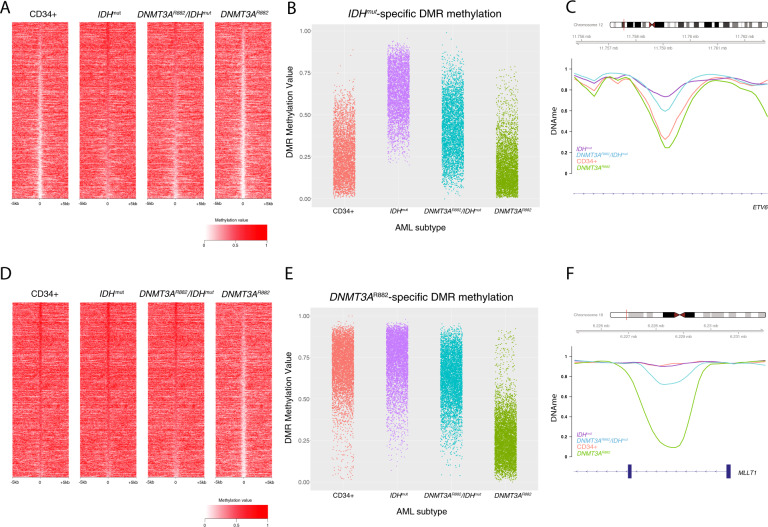
*DNMT3A*^R882^/*IDH*^mut^ double mutant AMLs display an attenuated focal hypermethylation phenotype. **A** Locus heatmap of mean methylation at *IDH*^mut^ DMRs (rows) in *IDH1* or *IDH2* mutant, *DNMT3A*^R882^/*IDH*^mut^ double mutant, and *DNMT3A*^R882^ AMLs, and CD34+ cells. **B** Distribution of *IDH*^mut^-specific DMR methylation levels by AML subtype. **C** Example *IDH*^mut^-specific DMR locus within the *ETV6* gene demonstrating an intermediate methylation phenotype of double mutant samples with respect to *IDH*^mut^ and *DNMT3A*^R882^ mutant AMLs. **D** Methylation locus heatmap of average subtype methylation across *DNMT3A*^R882^ DMRs called vs. CD34+ cells in *IDH*^mut^, *DNMT3A*^R882^/*IDH*^mut^ double mutant, and *DNMT3A*^R882^ AMLs, and CD34+ cells. **E** Distribution of *DNMT3A*^R882^ DMR methylation levels by AML subtype. **F** Example *DNMT3A*^R882^ DMR locus within the *MLLT1* gene, demonstrating the hypomethylation phenotype of *DNMT3A*^R882^ mutant samples with respect to *IDH*^mut^ and *DNMT3A*^R882^/*IDH*^mut^ double mutant AML samples.

To assess whether de novo DNA methylation by DNMT3A contributes to *IDH*^mut^-associated hypermethylation, we analyzed methylation levels at *IDH*^mut^-specific DMRs in seven AML samples with co-occurring *IDH1* (*N* = 5) or *IDH2* (*N* = 2) and *DNMT3A*^R882^ mutations (R882 mutations have a more severe hypomethylation phenotype than other *DNMT3A* mutations [4, 39]). Interestingly, although *DNMT3A*^R882^/*IDH*^mut^ AMLs were still hypermethylated at *IDH*^mut^-specific DMRs, the degree of hypermethylation was diminished, with 67% of these regions having significantly lower DNA methylation levels than samples with *IDH* mutations alone (3024 of 4541 regions having a beta-binomial adjusted *P* < 0.05; see Fig. 4A–C, S3A). Similar findings were observed in seven additional *DNMT3A*^R882^/*IDH*^mut^ AML samples using methylation array data from the TCGA AML study [15] (Fig. S4B). To further characterize the extent of this interaction, we analyzed DNA methylation levels in *DNMT3A*^R882^/*IDH*^mut^ AML samples at hypomethylated DMRs in AMLs with the *DNMT3A*^R882^ allele [5]. Surprisingly, these regions remained nearly fully methylated in the *DNMT3A*^R882^/*IDH*^mut^ double mutant samples, with 93% of the regions having significantly higher methylation than AMLs with *DNMT3A*^R882^ alone (4209 of 4541 regions having a beta-binomial adjust *P* < 0.05; see Fig. 4D–F, Fig. S4C). Similar findings were observed in the AML TCGA data [15] (Fig. S4D), strongly suggesting that DNMT3A-mediated methylation and TET-mediated demethylation occur at the same places in the genome.

### *IDH*^mut^-specific hypermethylated DMRs are enriched for enhancers

**Fig. 5:**
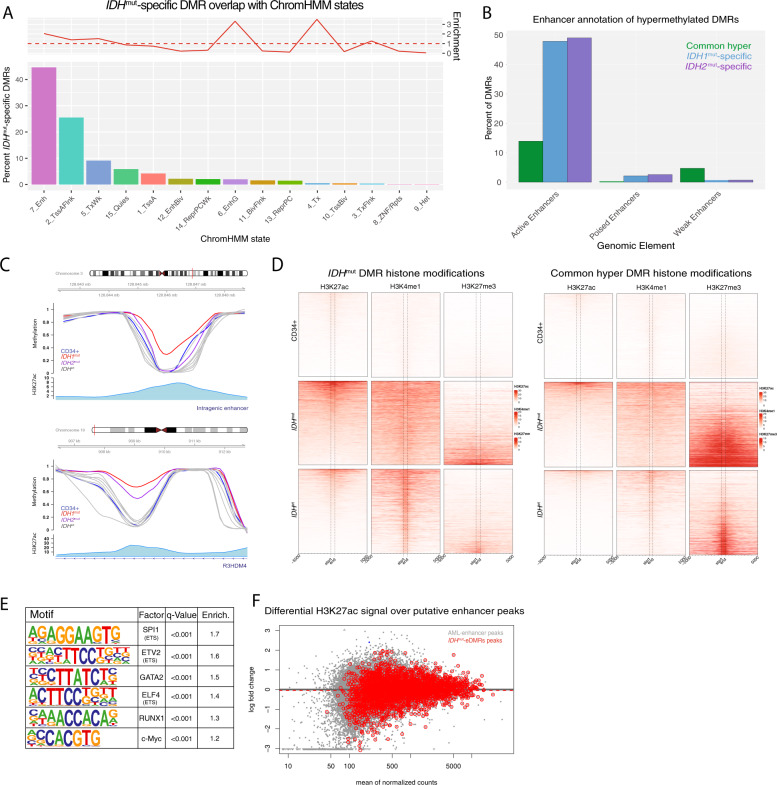
*IDH*^mut^-specific DMRs are enriched for putative enhancers. **A** Distribution of ChromHMM chromatin states from CD34+ cells represented in *IDH*^mut^-specific DMRs. Enrichment of chromatin states within *IDH*^mut^-specific DMRs is shown with respect to the frequency of states overlapping regions of common CpG island hypermethylation. **B** Enhancer-based annotation of common hypermethylated regions, *IDH1*^mut^, and *IDH2*^mut^ DMRs, where DMRs overlapping an H3K27ac peak alone or in combination with H3K4me1 were called active enhancers, those overlapping a H3K27ac peak in combination with H3K27me3 were called poised enhancers, and those overlapping a H3K4me1 alone were called  weak enhancers. **C** Examples of intragenic and genic enhancer regions exhibiting *IDH1*^mut^, *IDH2*^mut^, or *IDH1/2*^mut^ hypermethylation compared with CD34+ cells and other AML subtypes. **D** Heatmap of enhancer histone modifications and heterochromatin modifications over *IDH*^mut^-specific DMRs (left) and generic hypermethylation (right) in CD34+ cells (*N* = 4 H3K27ac, *N* = 7 H3K3me1, and *N* = 7 H3K27me3), *IDH*^mut^ AML (*n* = 3), and *IDH*^wt^ AML samples (*N* = 9 H3K27ac, *N* = 10 H3K3me1, and *N* = 24 H3K27me3). **E** HOMER motif enrichment analysis of *IDH1/2*^mut^-specific DMRs with respect to a background set of generically hypermethylated regions. **F** Differential active enhancer signal (H3K27ac) for all AML-associated putative enhancers (black points) compared with putative enhancers intersecting an *IDH1/2*^mut^-specific DMR (red points).

We next asked whether *IDH*^mut^-specific DMRs were associated with certain chromatin states. Annotation of these DMRs with chromatin states in CD34+ cells [36] demonstrated that 44% occurred in enhancers, which was a twofold enrichment over regions commonly hypermethylated (Fig. 5A). This enrichment was not observed in analyses on DMRs identified in other AML subtypes (Fig. S5A–C). We further defined this association using ChIP-seq peaks for active, weak, and poised enhancers using ChIP-seq data for H3K27ac, H3K4me1, and H3K27me3 modifications from 16 primary AML samples, including two with *IDH* mutations. This demonstrated that 47% of the *IDH*^mut^ DMRs overlapped an active enhancer, compared to 3% and 1% that overlapped poised and weak regions, respectively (Fig. 5B–D). In comparison, commonly hypermethylated regions showed less overlap with active enhancers (13% of DMRs) and greater intersection with repressive H3K27me3 marks (Fig. 5D). Analysis of *IDH*^mut^-specific DMRs for transcription factor (TF) binding motifs identified binding sites for hematopoietic-associated TFs, including *SPI1*, *RUNX1*, and *MYC* (Fig. 5E), further supporting the occurrence of *IDH*^mut^-specific hypermethylation in regions with potential regulatory activity. However, quantitative analysis of H3K27ac signal over these regions in samples with and without *IDH* mutations did not identify appreciable differences (*P* = 0.24, Fig. 5F), suggesting that hypermethylation does not modify H3K27ac levels within these regions.

### *IDH*^mut^-specific DMRs occur in enhancers that form direct interactions with highly expressed genes in AML cells

**Fig. 6:**
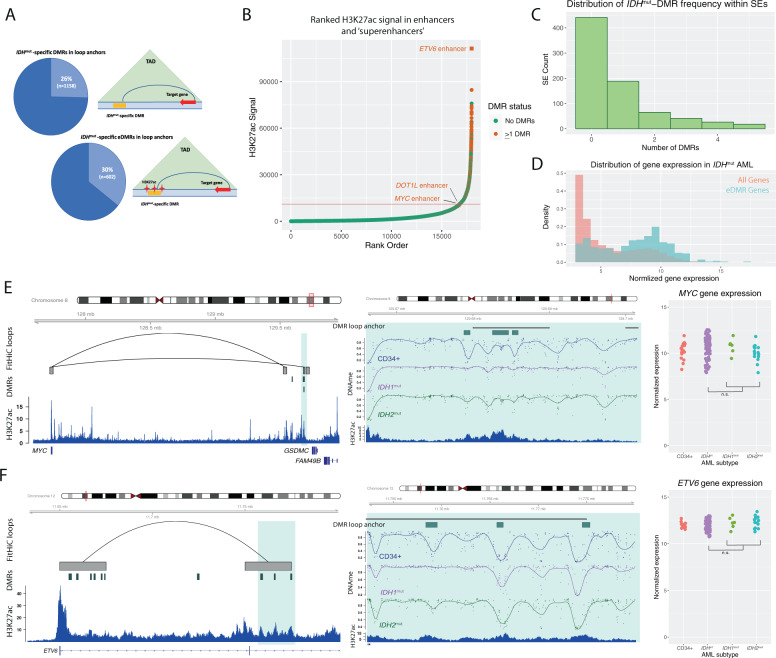
*IDH*^mut^-specific DMRs are enriched in superenhancers and interact with highly expressed genes in AML. **A** Schematic of a DMR and an enhancer-associated DMR (eDMR) and their interaction with target genes based on intersection HiC-defined genome loops. **B** Rank ordered enhancer regions based on H3K27ac signal in a representative *IDH*^mut^ AML sample, annotated by the presence of overlapping *IDH*^mut^-specific DMRs (absence of DMRs indicated by green points, greater than one DMR indicated by orange points) and computationally-defined “superenhancer” (above the red line). Enhancers of specific hematopoietic genes are labeled. **C** Distribution of the number of *IDH*^mut^-specific DMRs overlapping a set of AML consensus superenhancers from H3K27ac data from four primary samples (*N* = 779). **D** Distribution of normalized gene expression values for all expressed genes (orange histogram) and a set of 750 eDMR target genes (blue histogram) in *IDH*^mut^ AML samples. **E** Example *IDH*^mut^-eDMR locus displaying interactions with the *MYC* promoter. A zoomed-in view of the locus demonstrates focal enhancer hypermethylation in *IDH1*^mut^ (purple) and *IDH2*^mut^ (green) samples compared with CD34+ cells (blue). Normalized *MYC* expression is shown for 17 CD34+ cord blood cell samples, 6 and 14 *IDH1*^mut^ and *IDH2*^mut^ samples, and 91 *IDH*^wt^ samples. **F** Example *IDH*^mut^-DMR locus in a candidate enhancer that displays robust interactions with the *ETV6* promoter. A zoomed-in locus view demonstrates focal enhancer hypermethylation in *IDH1*^mut^ (purple) and *IDH2*^mut^ (green) samples compared with CD34+ cells (blue). Normalized *ETV6* expression is shown for CD34+ cells, *IDH1*^mut^, and *IDH2*^mut^ samples, and *IDH*^wt^ samples (see **E** for sample sizes).

We next asked whether enhancers with *IDH*^mut^ DMRs could be involved in controlling gene expression relevant for AML pathogenesis. To directly link these enhancers to their target genes, we analyzed 3D genome interactions generated using in situ HiC from both normal CD34+ cells [32] and three primary AML samples [31] (all were wild type for *DNMT3A*, *IDH1*, *IDH2*, and *TET2*). This analysis demonstrated that 26% (1158/4541) of all *IDH*^mut^-specific DMRs and 30% (602/2000) of the DMRs in putative enhancers overlapped the “loop anchor” of a genome interaction (Fig. 6A, Fig. S6A). *IDH*^mut^ DMRs in these loop anchors were highly enriched in “superenhancers”, with between 37 and 39% of superenhancers defined in three primary *IDH*^mut^ AML samples containing at least one *IDH*^mut^-specific DMR (Fig. 6B, C, Fig. S6B, C). We next analyzed gene expression in 750 genes with promoters that formed 3D interactions with *IDH*^mut^-specific DMRs using RNA-seq data from 179 AML samples from the TCGA AML study. This showed that the genes linked to *IDH*^mut^-specific DMRs were highly expressed, with 68% of these genes ranked in the top 25th percentile of gene expression (Fig. 6D, Fig. S6D). Further analysis of 3D genome interactions containing *IDH*^mut^-specific DMRs identified known and novel enhancers of genes important in hematopoiesis and AML, including an enhancer of *MYC* [40–42] (Fig. 6E), and previously unreported putative enhancers that form interactions with *ETV6* (Fig. 6F), *DOT1L*, and *SRSF3* (Fig. S6E, F). Although we did not observe significant changes in expression of these genes between *IDH*^mut^ and *IDH*^wt^ AMLs, their high expression in AML samples and CD34+ cells was consistent with the enrichment of *IDH*^mut^-specific DMRs in enhancers of active genes (Fig. 6D–F).

## Discussion

Recurrent gain-of-function *IDH* mutations increase DNA methylation, but the genomic locations and functional consequences of these changes have not previously been clearly defined. Our analysis of WGBS data from primary AML samples shows that methylation changes caused by these mutations are not widespread but instead manifest as thousands of focal regions that are uniquely hypermethylated compared to normal CD34+ cells and AML cells without *IDH* mutations. These regions had lower CpG density and fewer CGIs than loci that are commonly hypermethylated in AML, suggesting that *IDH*^mut^-associated hypermethylation is caused by a distinct mechanism. The *IDH2*^mut^ AMLs in our dataset had less pronounced hypermethylation than those with *IDH1* mutations, but both were hypermethylated at a highly overlapping set of loci. AMLs with biallelic inactivating *TET2* mutations had a far less dramatic methylation phenotype, although many of the hypermethylated DMRs identified in these samples overlapped an *IDH*^mut^-specific DMR. Further, oxidative bisulfite sequencing demonstrated increased levels of 5hmC in these regions in AML samples that were wild type for *TET2*, *IDH1,* and *IDH2*; 5hmC levels were significantly lower in *IDH*^mut^ or *TET*2^mut^ samples in these regions, providing evidence that these mutations cause increased DNA methylation by impairing TET-mediated DNA demethylation. Regions with *IDH*^mut^-specific hypermethylation were enriched for active enhancers, many of which formed direct interactions with highly expressed AML genes, including *MYC* and *ETV6*. Although increased methylation at these loci was not associated with repressed chromatin or lower gene expression in *IDH*^mut^ AML samples, this finding demonstrates that *IDH*^mut^-associated hypermethylation affects the regulatory sequences of genes that may contribute to AML pathogenesis.

This study adds new context to the dynamics of de novo DNA methylation and active demethylation pathways in normal hematopoietic cells and in AML. The fact that *IDH*^mut^-associated hypermethylation occurs at regions with low levels of DNA methylation in normal CD34+ cells suggests that de novo DNA methylation and TET-mediated demethylation are both active in these regions, despite their low steady-state methylation levels. This is supported by the observation that AML samples with co-occurring *IDH* and *DNMT3A*^*R882*^ mutations show significantly attenuated hypermethylation, and that *IDH*^mut^-specific DMRs have high levels of 5hmC, which is produced from 5mC as a substrate. Remodeling of DNA methylation by these processes in specific regions has been reported previously in studies of embryonic stem cells, which have shown that methylation and active demethylation are in equilibrium at many loci [1, 2], and may be maintained by the occupancy of methylation and demethylation complexes [43]. Our analysis suggests this equilibrium also exists in normal hematopoietic stem/progenitor cells and is disrupted in the presence of mutant *IDH* alleles, leaving de novo DNA methylation unopposed. The focal nature of *IDH*^mut^-associated hypermethylation implies that activity (or occupancy) of DNMT3A and TET enzymes is not diffuse and may instead be targeted to specific genomic regions. The genomic or epigenetic features directing this activity are unclear [44], but the enrichment of *IDH*^mut^ DMRs in active enhancers suggests that components of active chromatin may recruit methylation and demethylation machinery. The convergence of these processes at enhancers could provide clues as to why mutations with opposite effects on DNA methylation both contribute to AML development, perhaps via dysregulation of common target genes.

Our analysis of 3D genome interactions involving *IDH*^mut^-specific DMRs found that these sequences directly interact with genes that are highly expressed in hematopoiesis and AML (e.g., *MYC* and *ETV6*). Contrary to the canonical relationship between DNA methylation and activity, hypermethylation in the *IDH*^mut^ AML samples does not appear to repress either the enhancer elements or the expression of their target genes. Other regulatory factors may therefore be dominant to DNA methylation at these loci, and result in persistently high gene expression. It is also possible that regions of active chromatin, such as enhancers (and superenhancers), have high rates of methylation turnover, and are therefore more susceptible to perturbations in methylation and demethylation [1, 2]. Focal hypermethylation may occur in DNA elements bound by factors that contribute to “fine-tuning” these enhancers in specific cellular or developmental contexts, but that do not drive their activity in AML cells. Additional studies will be necessary to understand whether enhancer hypermethylation is a consequence of decreased occupancy of these modulating factors [45], or whether it directly prevents proper regulation in ways that contribute to AML development.

## Supplementary information

Figure S1

Figure S2

Figure S3

Figure S4

Figure S5

Figure S6

Supplemental Material

Table S1

Table S2

Table S3

## Data Availability

All raw data from primary AML samples presented in this study are available in dbGaP (accession number phs000159). Processed data from this study are available for public download at the following site: https://wustl.box.com/v/wilsonIDHmethylation.
