# Supplementary figures and images for: Focal disruption of DNA methylation dynamics at enhancers in IDH-mutant AML cells
Source: Leukemia. 2021 Dec 6;36(4):935–45. doi: 10.1038/s41375-021-01476-y (PMC8979817; doi:10.1038/s41375-021-01476-y)

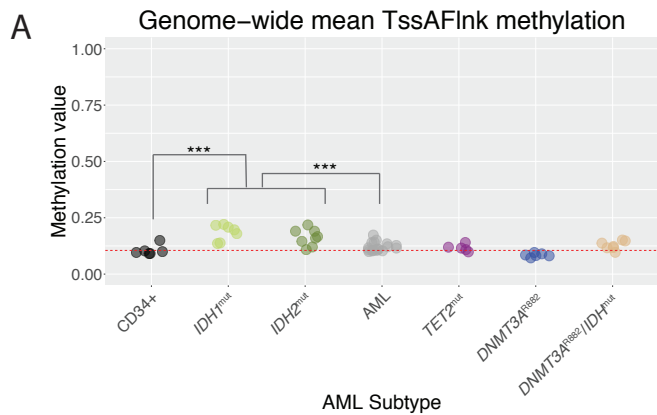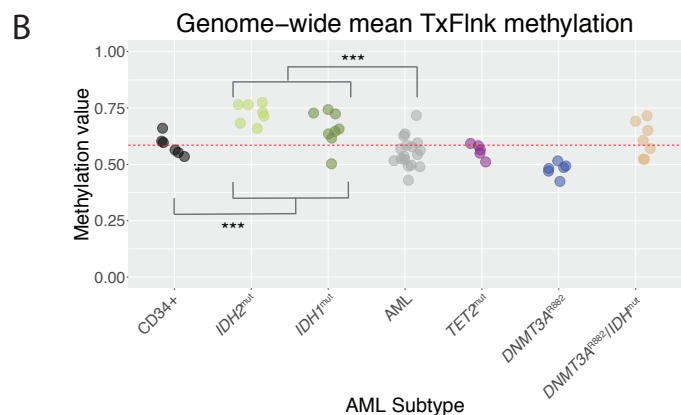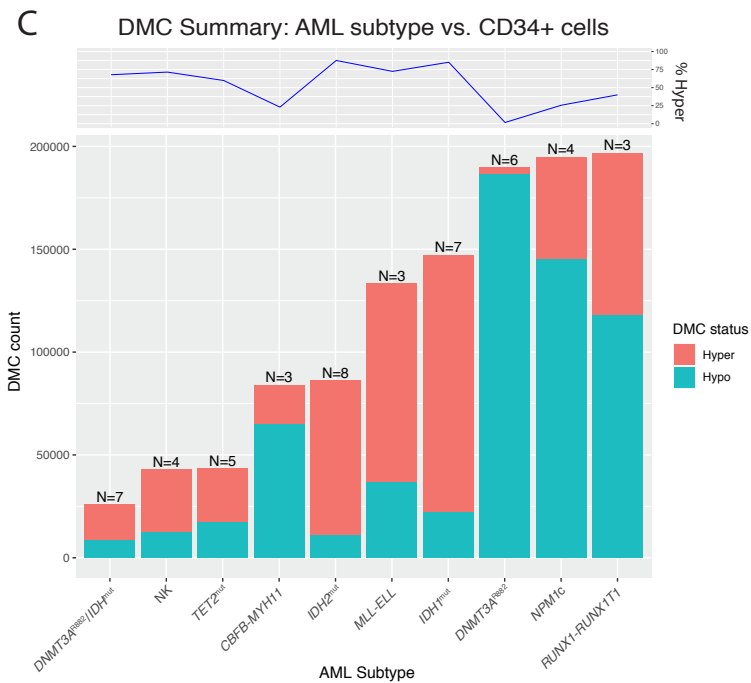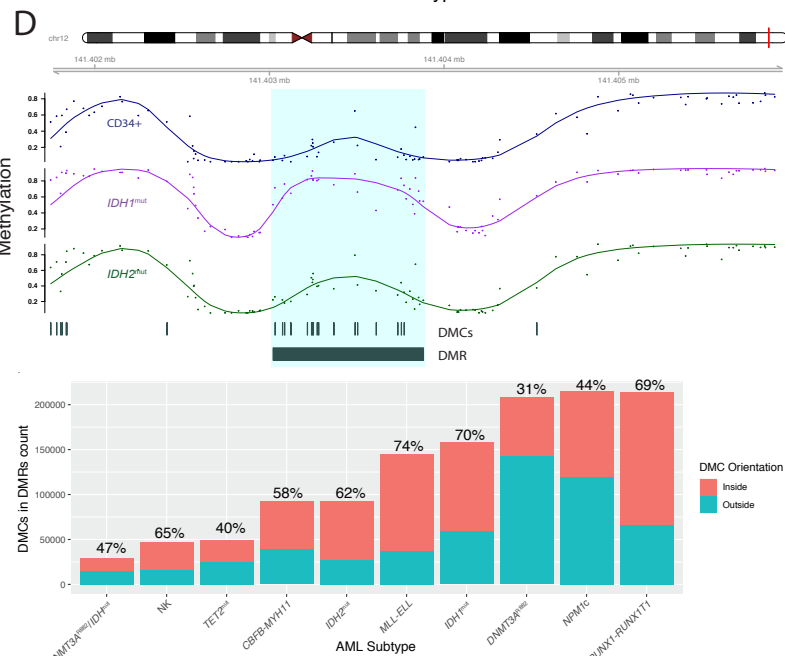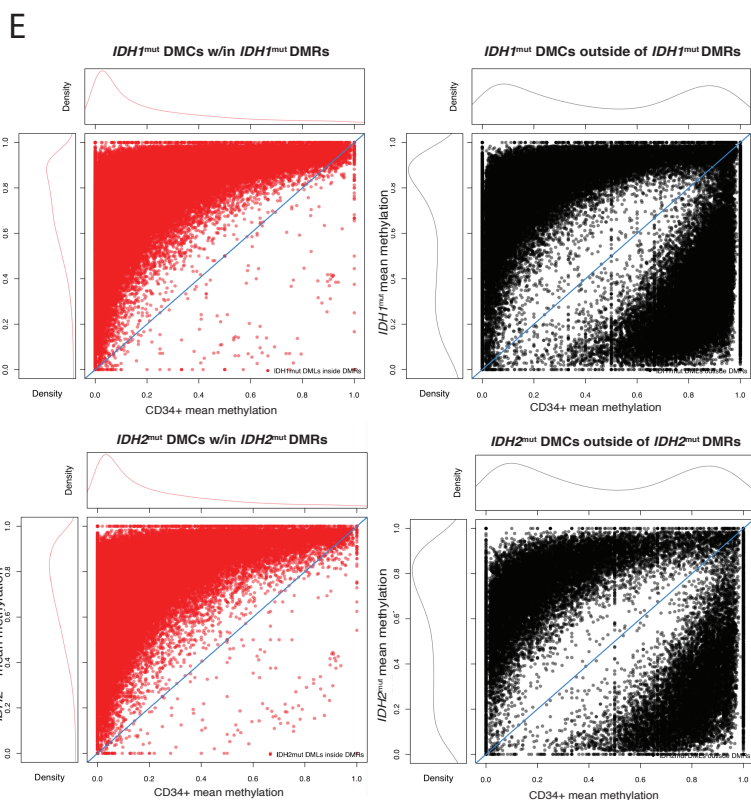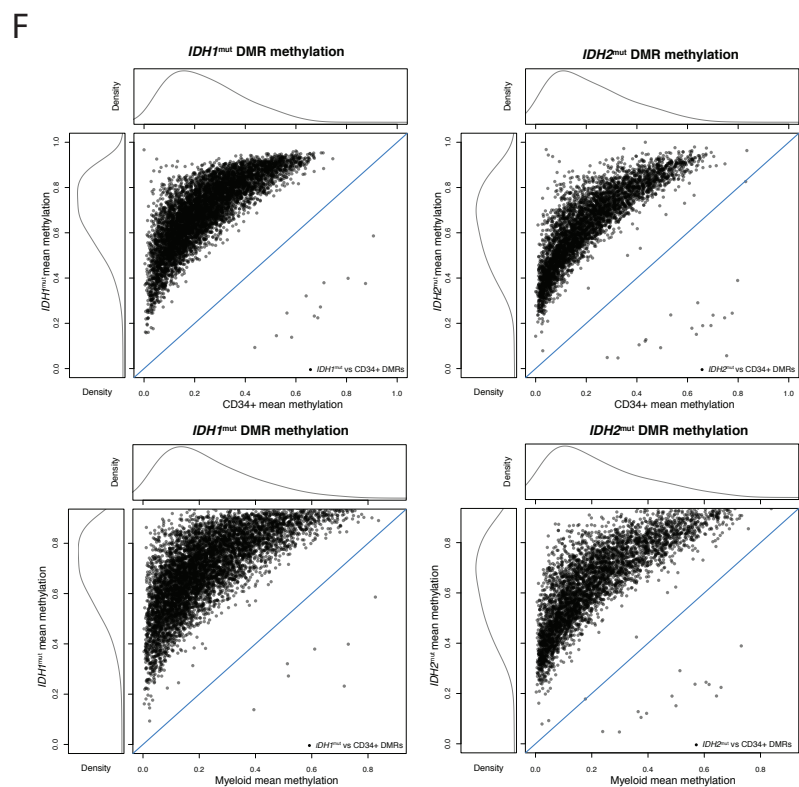

Supplement: Supplementary file 1 — Figure S1 [file 41375_2021_1476_MOESM1_ESM.pdf]

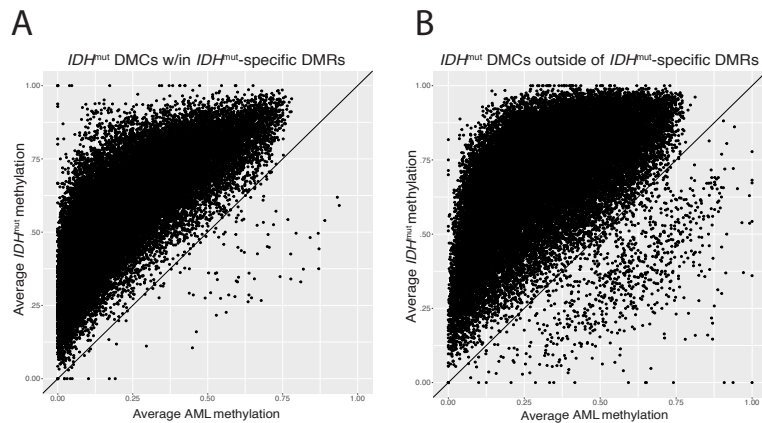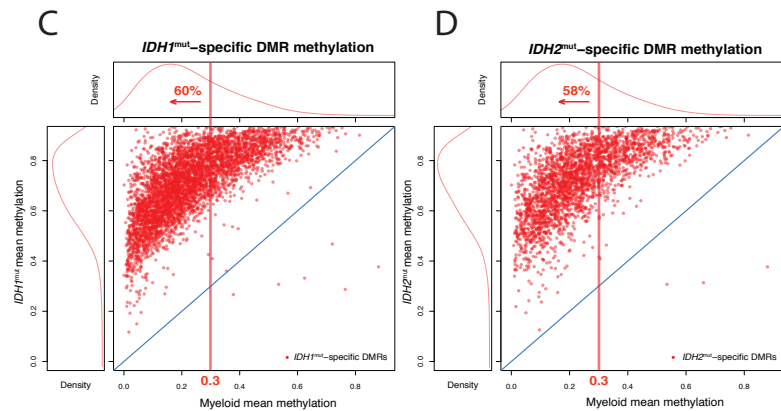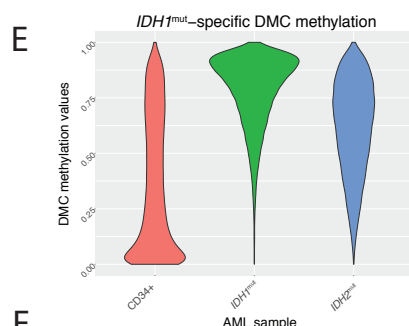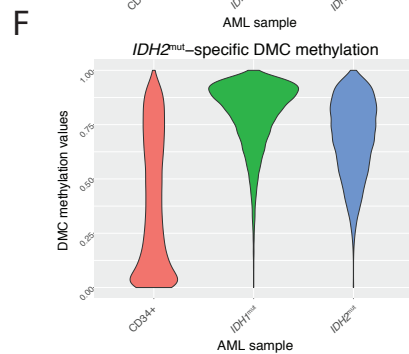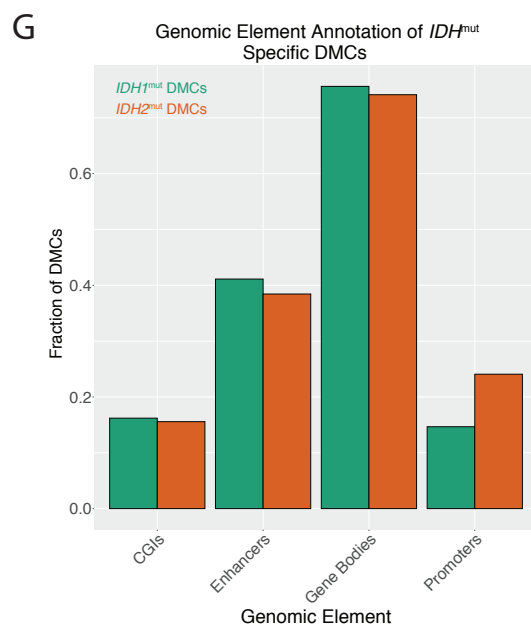

Supplement: Supplementary file 2 — Figure S2 [file 41375_2021_1476_MOESM2_ESM.pdf]

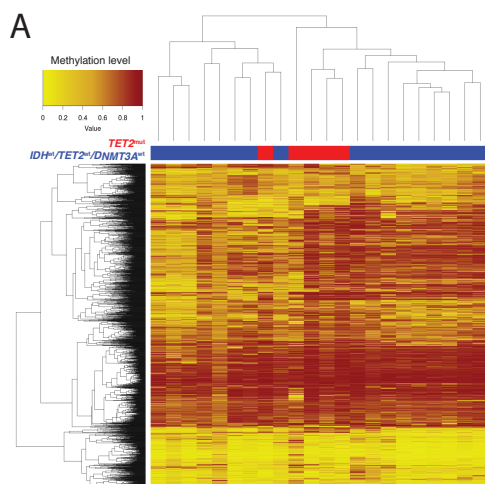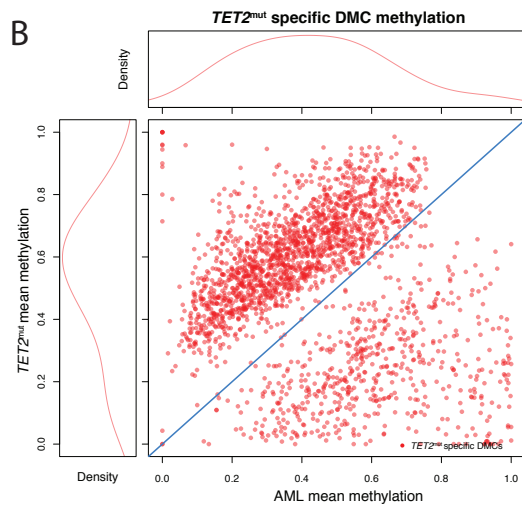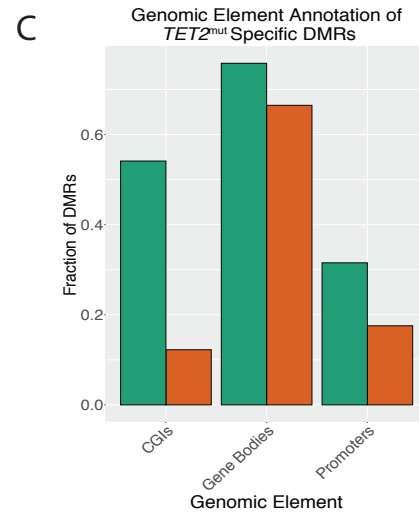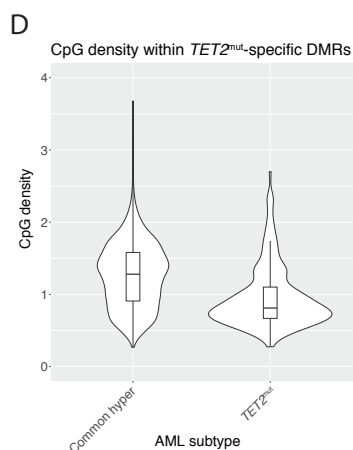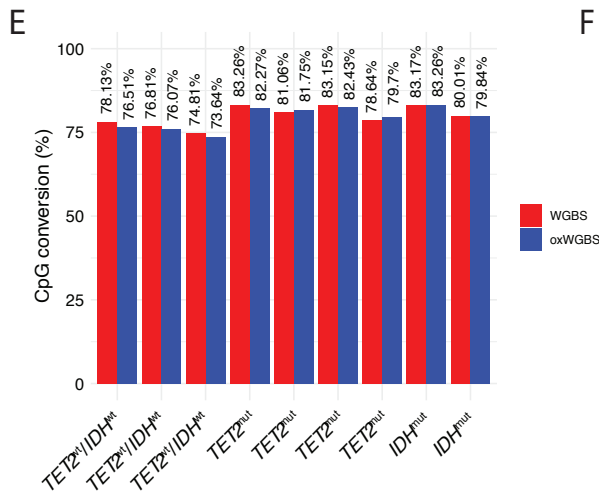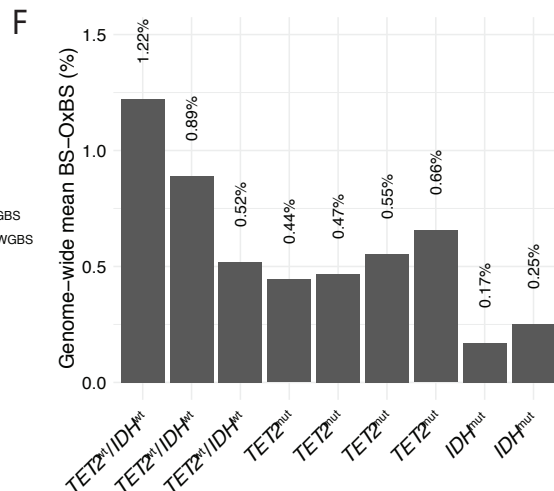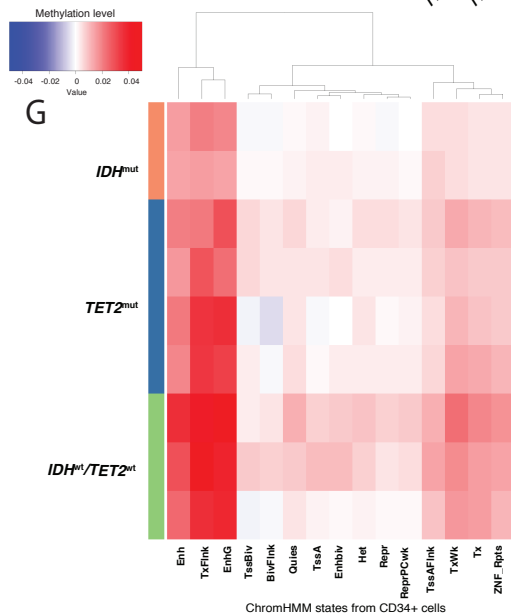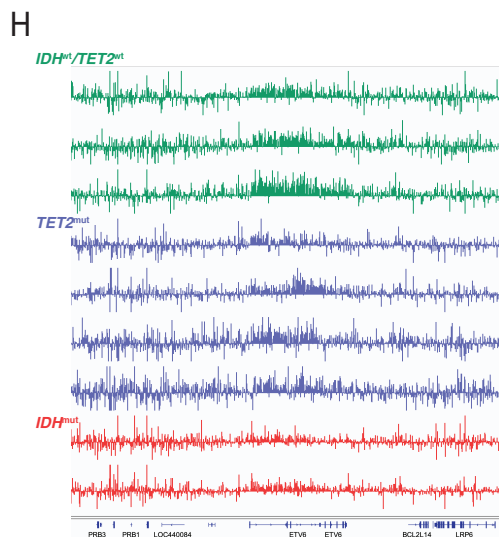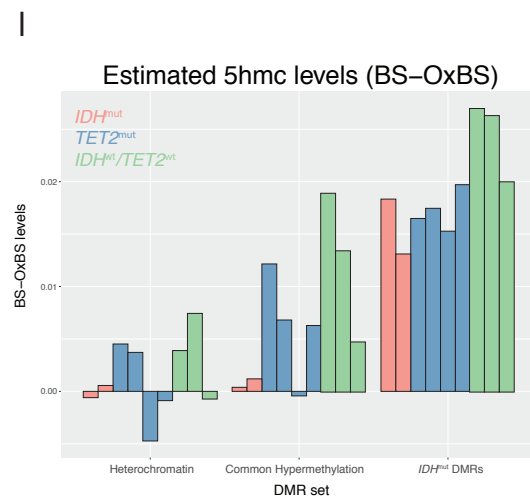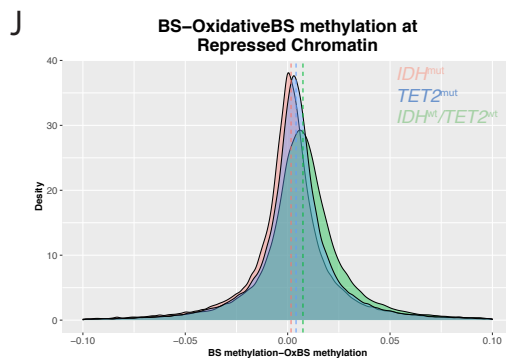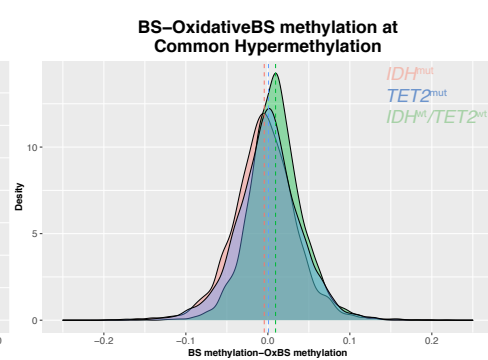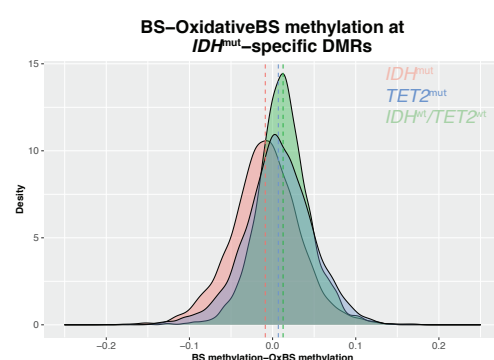

Supplement: Supplementary file 3 — Figure S3 [file 41375_2021_1476_MOESM3_ESM.pdf]

A

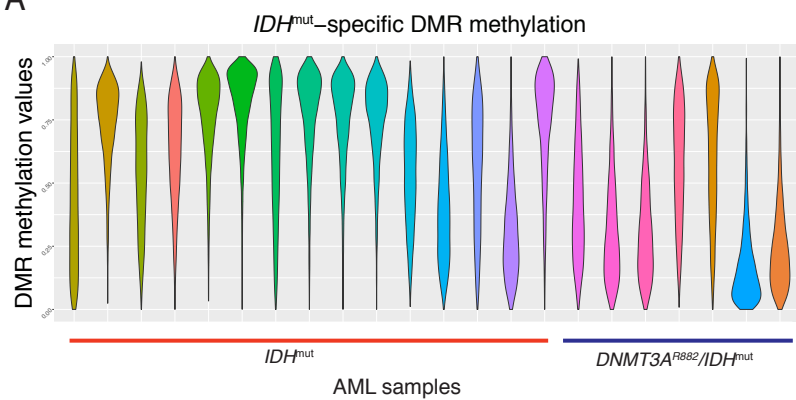

B

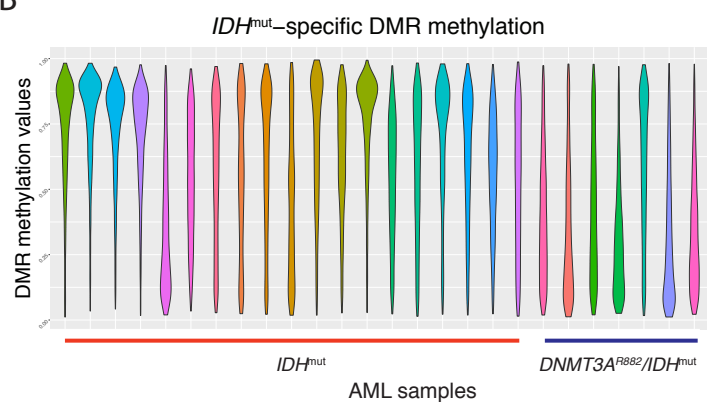

C

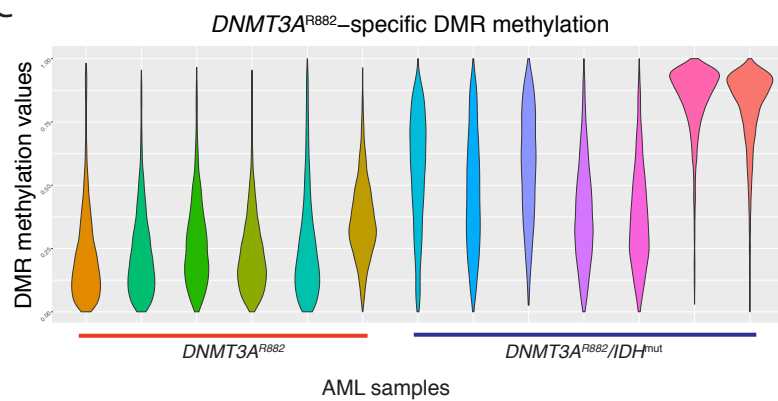

D

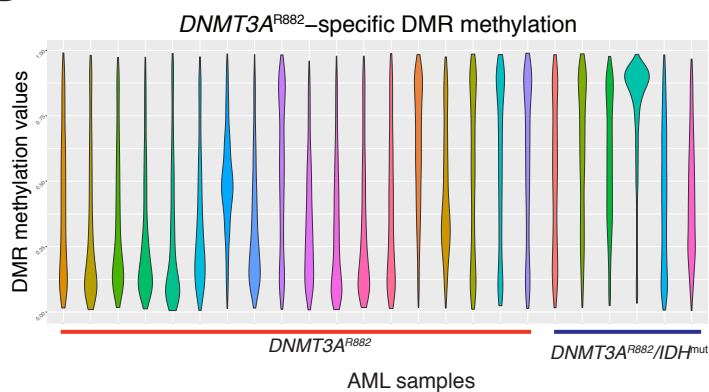

E

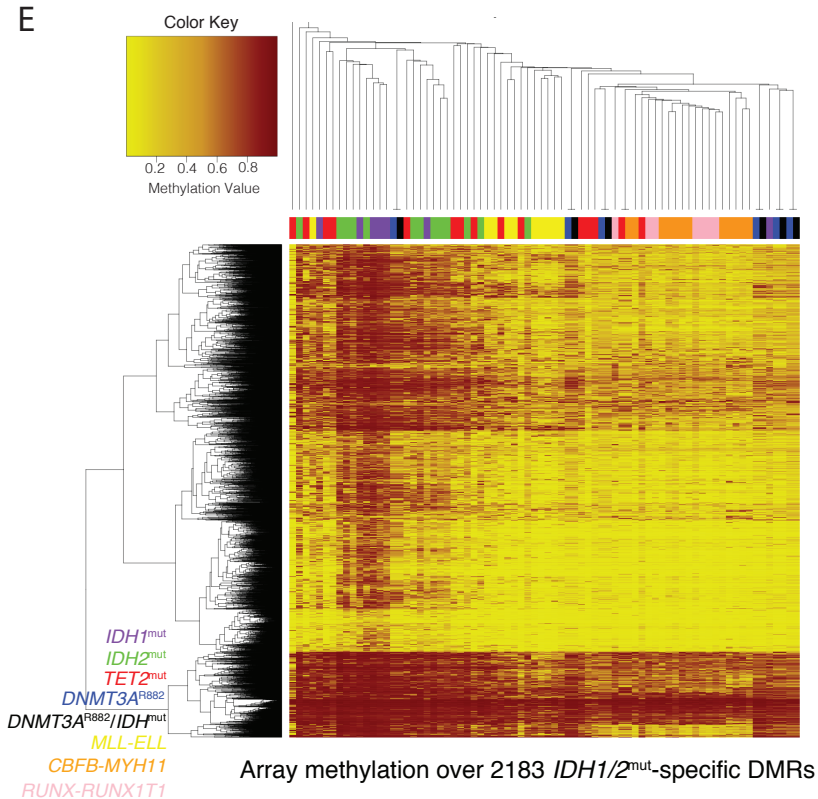

F

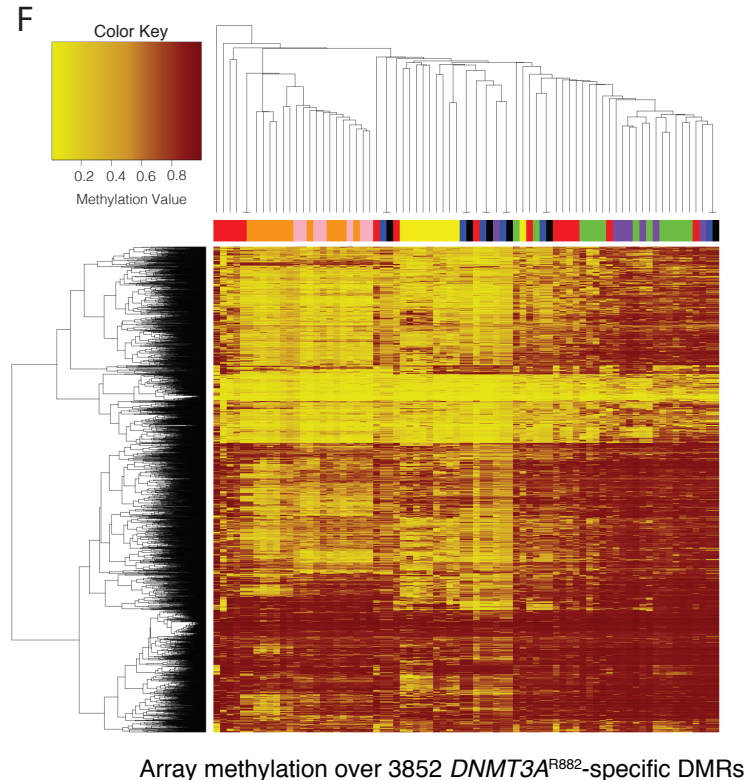

Supplement: Supplementary file 4 — Figure S4 [file 41375_2021_1476_MOESM4_ESM.pdf]

A

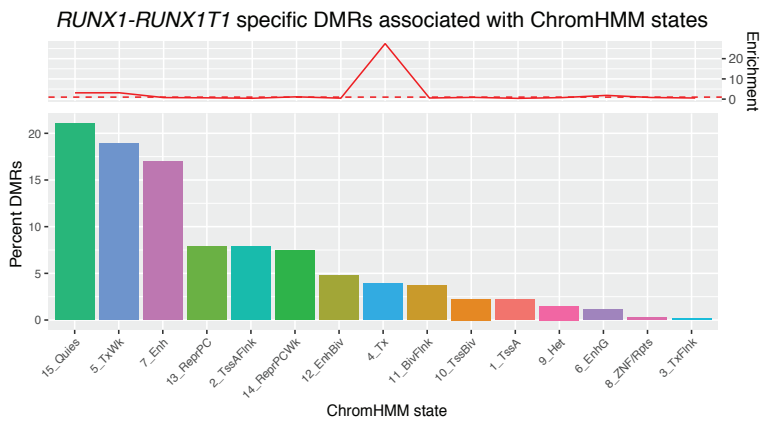

B

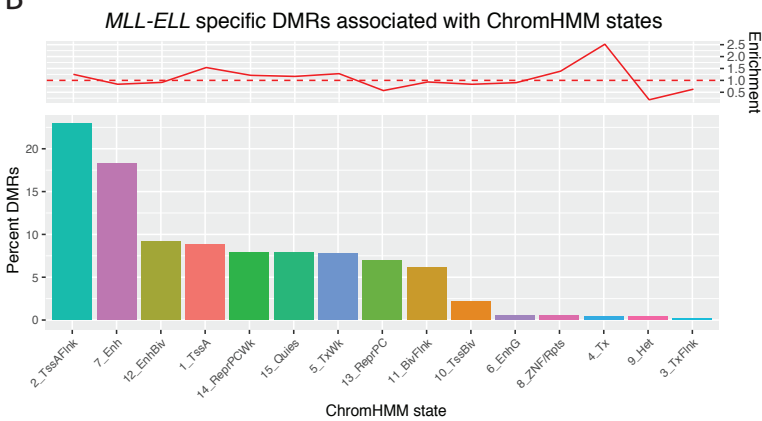

C

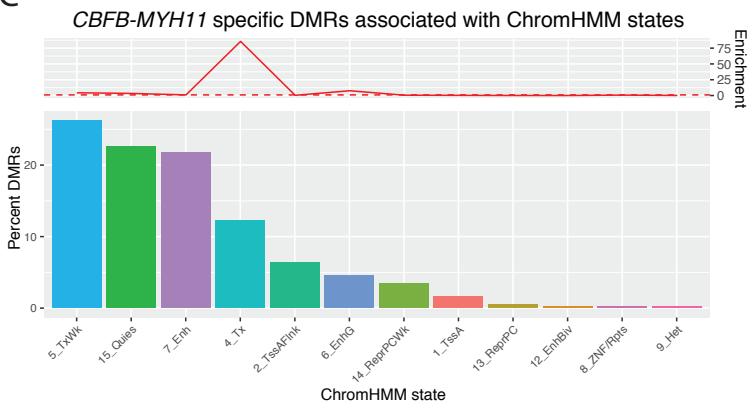

Supplement: Supplementary file 5 — Figure S5 [file 41375_2021_1476_MOESM5_ESM.pdf]

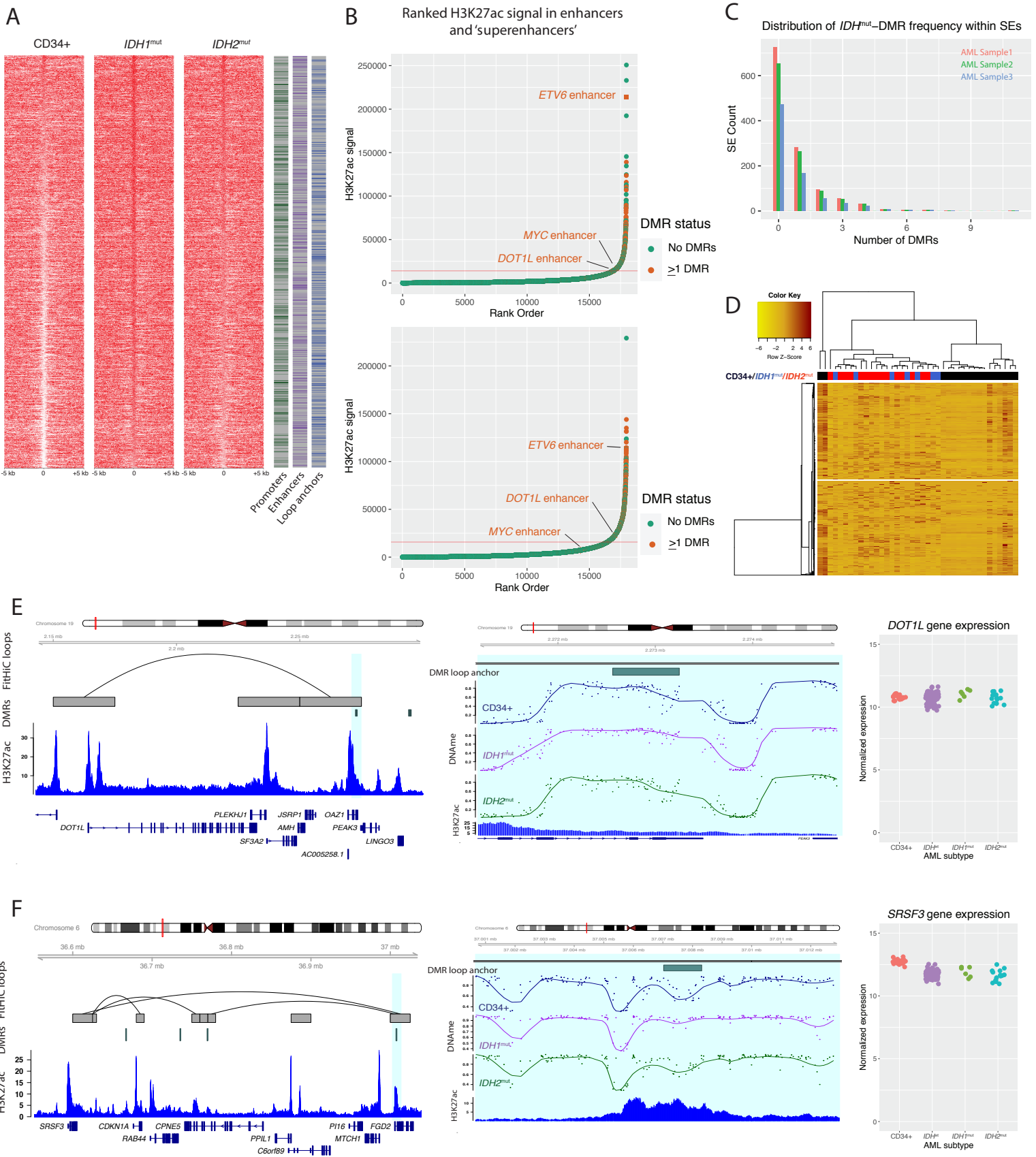

Supplement: Supplementary file 6 — Figure S6 [file 41375_2021_1476_MOESM6_ESM.pdf]
