# Supplementary material for: Focal disruption of DNA methylation dynamics at enhancers in IDH-mutant AML cells
Source: Leukemia. 2021 Dec 6;36(4):935–45. doi: 10.1038/s41375-021-01476-y (PMC8979817; doi:10.1038/s41375-021-01476-y)
Supplement: Supplementary file 7 — Supplemental Material [file 41375_2021_1476_MOESM7_ESM.docx]

**Figure S1.** *IDH*^mut^ AMLs exhibit the greatest proportion of focal increases in methylation compared with CD34+ cells. A. DMC summary for individual AML mutational subtypes compared with CD34+ cells. DMC differential methylation status is classified as either hypermethylated (orange bars) or hypomethylated (teal bars) with respect to CD34+ cells. B. Top panel: Example locus with a focal enrichment of dmCpGs where the highlighted cytosines were identified as constituents of a DMR with statistically increased methylation in *IDH*^mut^ samples compared with CD34+ cells. Bottom panel: Summary of DMCs localizing within/outside DMRs identified in individual subtypes vs. CD34+ cells. C-D. Average methylation levels across TssAFlnk (C) and TxFlnk (D) ChromHMM regions in CD34+ cells (N=6) and AML subtypes (*IDH1*^mut^ or *IDH2*^mut^, n=15; *TET2*^mut^, n=5; *DNMT3A*^R882^, n=6; *DNMT3A*^R882^/*IDH*^mut^, n=7; normal karyotype with *NPM1c* and wild-type *IDH1*, *IDH2*, *TET2*, and *DNMT3A,* n=4; Normal karyotype with wild-type *NPM1*, *IDH1*, *IDH2*, *TET2*, and *DNMT3A*, n=4; *CBFB-MYH11*, n=3; *KMT2A-ELL*, n=3; *RUNX1-RUNX1T1*, n=3). E. Distribution of mean methylation values across *IDH*^mut^ DMCs localizing within (left panels) or outside (right panels) *IDH1*^mut^ (top panels) and *IDH2*^mut^ (bottom panels) DMRs in *IDH*^mut^ AML samples vs. CD34+ HSPCs. F. Distribution of mean methylation values across *IDH1*^mut^ (top panels) and *IDH2*^mut^ (bottom panels) DMRs in *IDH*^mut^ AML samples vs. normal myeloid cells (n=3 promyelocyte samples; n=3 polymorphonuclear leukocyte samples; n=2 monocyte samples).

**Figure S2.** *IDH*^mut^-specific DMCs exhibit similar methylation patterns and genomic annotations as *IDH*^mut^-specific DMRs. A-B. Distribution of mean methylation across *IDH*^mut^ DMCs localizing within (A) or outside of (B) *IDH*^mut^-specific DMRs in *IDH*^mut^ samples vs. all other *IDH*^wt^/*TET2*^wt^/*DNMT3A*^wt^ AMLs. C-D. Distribution of mean methylation values across *IDH1*^mut^-specific (C) and *IDH2*^mut^-specific (D) DMRs in *IDH*^mut^ AML samples vs. normal myeloid cells (n=3 promyelocyte samples; n=3 polymorphonuclear leukocyte samples; n=2 monocyte samples). E-F. Violin plots of methylation levels across all *IDH1*^mut^-specific (E) and *IDH2*^mut^-specific (F) DMCs in CD34+ cells, *IDH1*^mut^ samples, and *IDH2*^mut^ samples. G. Fraction of *IDH1*^mut^-specific DMCs and *IDH2*^mut^-specific DMCs overlapping functional genomic elements including CpG islands (CGIs), enhancers, gene bodies, and promoters.

**Figure S3.** *TET2*^mut^-associated hypermethylation is distinct from canonical CpG-island hypermethylation and is consistent with regions of increased TET2 hydroxymethylation activity in *TET2*^wt^ cells**.** A. Hierarchical clustering of *TET2*^mut^ AMLs with all other *IDH*^wt^, *TET2*^wt^, and *DNMT3A*^wt^ AMLs at the set of *TET2*^mut^ vs. CD34+ DMRs. Column annotation represents mutational classification by sample. B. Distribution of *TET2*^mut^-specific DMC mean methylation in *TET2*^mut^ samples vs. the set of *IDH*^wt^/*TET2*^wt^/*DNMT3A*^wt^ AMLs. C. Percent overlap of generic AML-associated hypermethylation and *TET2*^mut^- specific DMRs with defined genomic annotations. D. Distribution of CpG density across the set of commonly hypermethylated CpG islands and *TET2*^mut^- specific DMRs. E. CpG conversion rate of 9 paired whole-genome bisulfite and oxidative bisulfite prepared libraries (n=2 for *IDH^mut^* ; n=4 for *TET2*^mut^; n=3 for *IDH*^wt^/*TET2*^wt^). F. Genome-wide average 5hmc levels across ~10.6 million CpGs with > 10x coverage in each of the paired samples, as calculated by subtracting oxidative bisulfite levels from bisulfite levels. G. Hierarchical clustering of mean 5-hmC levels across 15 chromatin states defined in CD34+ cells for individual patient samples. Columns represent chromatin states and rows represent individual patient samples. Vertical color blocks indicate *IDH* and *TET2* mutation status of individual samples. H. Example locus encompassing the *ETV6* gene with diminished 5hmc levels in two *IDH*^mut^ samples (red tracks) and four *TET2*^mut^ samples (blue tracks) compared with three *IDH*^wt^/*TET2*^wt^ samples (green track). I. Mean 5-hmC levels in *IDH*^mut^, *TET2*^mut^, and *IDH*^wt^/*TET2*^wt^ patient samples at heterochromatic regions, commonly hypermethylated regions, and IDHmut-specific DMRs. J. Distribution of estimated 5hmc levels across 4008 *IDH*^mut^ – specific DMRs, 4586 commonly hypermethylated regions in AML, and ~105,500 heterochromatic regions summarized by mutation.

**Figure S4.** Individual samples with *IDH* mutations alone and in combination with *DNMT3A*-*R882* exhibit group level methylation trends at *IDH*^mut^-specific and *DNMT3A-R882* DMRs. A. Methylation values across *IDH*^mut^-specific DMRs in a set of 15 *IDH*^mut^ samples (red underline) and 7 *DNMT3A*^R882^/*IDH* doubly mutant samples (blue underline) assayed with WGBS. B. Methylation values across *IDH*^mut^-specific DMRs in a set of 20 *IDH*^mut^ samples (red underline) and 6 *DNMT3A*^R882^/*IDH* double mutant samples (blue underline) assayed with methylation array. C. Methylation value across *DNMT3A*^R882^ DMRs in a set of 6 *DNMT3A*^R882^ samples (red underline) and 7 *DNMT3A*^R882^/*IDH* doubly mutant samples (blue underline) assayed with WGBS. D. Methylation values across *DNMT3A*^R882^ DMRs in a set of 18 *DNMT3A*^R882^ samples (red underline) and 6 *DNMT3A*^R882^/*IDH* double mutant samples (blue underline) assayed with methylation arrays. E. Hierarchical clustering of CpG methylation values contained within 2183 *IDH*^mut^-specific DMRs in primary AML samples with *IDH1* (n=7), *IDH2* (n=13), *TET2* (n=15), and *DNMT3A*^R882^ (n=6), and co-occurring *DNMT3A*^R882^/*IDH* (n=6), and also *MLL-ELL* (n=11), *CBFB-MYH11* (n=12), and *RUNX1-RUNX1T1* (n=7) fusions. F. Hierarchical clustering of CpG methylation values contained within 3852 *DNMT3A*^R882^ DMRs in primary AML samples with *IDH1* (n=7), *IDH2* (n=13), *TET2* (n=15), *DNMT3A*^R882^ (n=6), and co-occurring *DNMT3A*^R882^/*IDH* mutations (n=7), and also *MLL-ELL* (n=11), *CBFB-MYH11* (n=12), and *RUNX1-RUNX1T1* (n=7) fusions.

**Figure S5.** ChromHMM states are unique in subtype-specific DMRs for AMLs with canonical fusions and *DNMT3*A^R882^ mutations compared to *IDH*^mut^ AML. A. Percent overlap of 1921 RUNX1-RUNX1T1 DMRs with 15 ChromHMM chromatin states. B. Percent overlap of 276 *MLL-ELL* DMRs with 15 ChromHMM chromatin states. C. Percent overlap of 309 *CBFB-MYH11* DMRs with 15 ChromHMM chromatin states.

**Figure S6.** *IDH*^mut^-specific enhancer DMRs are enriched in ‘superenhancers’ and contact highly expressed genes in AML. A. Locus heatmap of mean subtype methylation across *IDH*^mut^-specific DMRs, including annotated overlaps with gene promoters (green), putative active enhancers (purple), and FitHiC loop anchors (blue). B. Representative rank-ordered analysis of H3K27ac marked enhancers in two *IDH*^wt^ AML samples annotated by enhancer and super-enhancer overlap with *IDH*^mut^-specific DMRs. C. Distribution of number of *IDH*^mut^-specific DMRs overlapping computationally defined ‘superenhancers’ in 3 *IDH*^mut^ AML samples. D. Hierarchical clustering of *IDH*^mut^ -eDMR target gene expression in *IDH1* (n=6), *IDH2* (n=14), and normal CD34+ cord blood cells (N=17, GSE48846). E. Example *IDH*^mut^ -eDMR locus displaying robust interactions with the *DOT1L* promoter. A zoomed in view of the locus demonstrates focal enhancer hypermethylation in *IDH1*^mut^ (purple) and *IDH2*^mut^ (green) samples compared with CD34+ cells (blue). Normalized *DOT1L* expression is shown for 17 CD34+ samples, 6 and 14 *IDH1*^mut^ and *IDH2*^mut^ samples, and 91 *IDH*^wt^ samples. F. Example of *IDH*^mut^ -eDMR locus displaying robust interactions with the *SRSF3* promoter. A zoomed in view of the locus demonstrates focal enhancer hypermethylation in *IDH1*^mut^ (purple) and *IDH2*^mut^ (green) samples compared with CD34+ cells (blue). Normalized *SRSF3* expression is shown for 17 CD34+ samples, 6 and 14 *IDH1*^mut^ and *IDH2*^mut^ samples, and 91 *IDH*^wt^ samples.
